# Draft Genome Sequence of Azospira sp. Strain I13, a Nitrous Oxide-Reducing Bacterium Harboring Clade II Type *nosZ*

**DOI:** 10.1128/genomeA.00414-18

**Published:** 2018-05-17

**Authors:** Toshikazu Suenaga, Tomo Aoyagi, Masaaki Hosomi, Tomoyuki Hori, Akihiko Terada

**Affiliations:** a Department of Chemical Engineering, Tokyo University of Agriculture and Technology, Koganei, Tokyo, Japan; b Environmental Management Research Institute, National Institute of Advanced Industrial Science and Technology (AIST), Tsukuba, Ibaraki, Japan

## Abstract

We report here a draft genome sequence of Azospira sp. strain I13 in the class Betaproteobacteria, a facultative anaerobic bacterium responsible for nitrous oxide (N_2_O) reduction. Deciphering this genome would pave the way for the use of Azospira sp. strain I13 to facilitate N_2_O consumption in a nitrogen-removing bioreactor emitting N_2_O.

## GENOME ANNOUNCEMENT

Nitrous oxide (N_2_O), a highly potent greenhouse gas causing ozone depletion, is utilized by microorganisms as an electron accepter in natural ecosystems and engineered systems, serving as a N_2_O sink (1, 2). It was recently reported that N_2_O-reducing bacteria are classified into two clade types based on sequences of a functional gene (*nosZ*) encoding N_2_O reductase (3, 4). Furthermore, distinct *nos* gene clusters with the two clades display divergent traits in terms of the gene expression, electron transfer, and N_2_O-reducing activity (4–6). The complete genome of Azospira suillum strain PS, a perchlorate-reducing bacterium, was previously reported (7, 8). Nevertheless, genome and physiological data about members of the genus Azospira remain scarce, especially regarding their role in nitrogen transformations. Recently, we isolated three strains of N_2_O-reducing bacteria by inoculating activated sludge in a municipal wastewater treatment plant with an enrichment reactor fed with sodium acetate and N_2_O as an electron donor and acceptor, respectively (T. Suenaga, T. Hori, S. Riya, M. Hosomi, B. F. Smets, and A. Terada, unpublished data). One of the isolates, Azospira sp. strain I13, has a high affinity for N_2_O and exhibits rapid recovery of N_2_O reduction activity from oxygen exposure-derived deterioration (9). The physiological traits are of promise for engineering applications in mitigating N_2_O emissions (9). We present here the draft genome sequence of Azospira sp. strain I13.

Azospira sp. strain I13 was aerobically grown using sodium acetate as a sole electron donor. Total nucleic acids were extracted by a phenol extraction method with chemical cell lysis and subsequently purified with cetyltrimethylammonium bromide. Then, DNA was purified by RNA decomposition with RNaseA (TaKaRa Bio, Inc., Japan). A paired-end DNA library (insert size, 250 to 500 bp) was prepared as previously reported (10). The library was sequenced using a MiSeq platform (Illumina, USA) and read with 700-fold genome coverage (10,672,103 250-bp paired-end reads). The acquired sequence, consisting of 26 scaffolds in total, was assembled using SOAPdenovo version 2.04 (11). The draft genome size of strain I13 was 3.79 Mb with a G+C content of 64.0%. The largest scaffold was 557 kb.

Fifty of the tRNA-encoding genes and three of the rRNA-encoding genes were identified by tRNAscan-SE version 1.3.1 (12) and RNAmmer version 1.2 (13), respectively. The draft genome sequence was annotated using the DDBJ Fast Annotation and Submission Tool (DFAST) (14), which yielded a total of 3,413 protein-coding DNA sequences. Azospira sp. strain I13 codes a clade II type *nosZ* gene. In addition, the strain possesses nitrogen metabolism-related genes for nitrate (dissimilatory NapAB), nitrite (*cd*_1_-containing NirS), nitric oxide, dissimilatory nitrite reduction, and nitrogen fixation. The draft genome sequence of Azospira sp. strain I13 will contribute to a comprehensive understanding of nitrogen metabolisms in natural environments and engineered systems.

### Accession number(s).

The draft genome of Azospira sp. strain I13 has been deposited as 26 scaffolds in DDBJ/EMBL/GenBank under the accession number BFBP00000000 (BFBP01000001 to BFBP01000026). The version described in this paper is the first version.
